# Demographics and outcomes of patients younger than 75 years undergoing aortic valve interventions in Rotterdam

**DOI:** 10.1007/s12471-024-01888-2

**Published:** 2024-08-20

**Authors:** Rik Adrichem, A. Maarten Mattace-Raso, Thijmen W. Hokken, Mark M. P. van den Dorpel, Marjo J. A. G. de Ronde, Mattie J. Lenzen, Paul A. Cummins, Isabella Kardys, Rutger-Jan Nuis, Joost Daemen, Jos A. Bekkers, Nicolas M. Van Mieghem

**Affiliations:** 1 Department of Cardiology, Erasmus University Medical Centre, Rotterdam, The Netherlands; 2 Department of Cardiothoracic Surgery, Erasmus University Medical Centre, Rotterdam, The Netherlands

**Keywords:** Transcatheter aortic valve implantation, Surgical aortic valve replacement, Risk assessment, Survival, Aortic valve disease

## Abstract

**Background:**

Transcatheter aortic valve implantation (TAVI) is considered a safe and effective alternative to surgical aortic valve replacement (SAVR) for elderly patients across the operative risk spectrum. In the Netherlands, TAVI is reimbursed only for patients with a high operative risk. Despite this, one fifth of TAVI patients are < 75 years of age. We aim to compare patient characteristics and outcomes of TAVI and SAVR patients < 75 years.

**Methods:**

This study included all patients < 75 years without active endocarditis undergoing TAVI or SAVR for severe aortic stenosis, mixed aortic valve disease or degenerated aortic bioprosthesis between 2015 and 2020 at the Erasmus University Medical Centre. Dutch authority guidelines were used to classify operative risk.

**Results:**

TAVI was performed in 292 patients, SAVR in 386 patients. Based on the Dutch risk algorithm, 59.6% of TAVI patients and 19.4% of SAVR patients were at high operative risk. There was no difference in 30-day all-cause mortality between TAVI and SAVR (2.4% vs 0.8%, *p* = 0.083). One-year and 5‑year mortality was higher after TAVI than after SAVR (1-year: 12.5% vs 4.3%, *p* < 0.001; 5‑year: 36.8% vs 12.0%, *p* < 0.001). Within risk categories we found no difference between treatment strategies. Independent predictors of mortality were cardiovascular comorbidities (left ventricular ejection fraction < 30%, atrial fibrillation, pulmonary hypertension) and the presence of malignancies, liver cirrhosis or immunomodulatory drug use.

**Conclusion:**

At the Erasmus University Medical Centre, in patients < 75 years, TAVI is selected for higher-risk phenotypes and overall has higher long-term mortality than SAVR. We found no evidence for worse outcome within risk categories.

**Supplementary Information:**

The online version of this article (10.1007/s12471-024-01888-2) contains supplementary material, which is available to authorized users.

## What’s new?

- Irrespective of age, younger patients undergoing transcatheter aortic valve implantation (TAVI) are vastly different from younger patients undergoing surgical aortic valve replacement (SAVR).
- Young TAVI patients are shown to have a higher mortality up to 5 years post-procedure.
- Although subgroup sample sizes are small, we found no evidence of a significant difference in treatment effect for patients undergoing TAVR or SAVR within the same risk stratum.
- Cardiovascular comorbidities and conditions such as malignancies and liver cirrhosis and the use of immunomodulatory drugs were independently associated with mortality.

## Introduction

Transcatheter aortic valve implantation (TAVI) has evolved from a last-resort treatment option for patients with severe aortic stenosis (AS) at prohibitive operative risk to a guideline-recommended treatment modality for elderly patients with severe AS across the entire operative risk spectrum [1–3]. Although age is an important variable in risk stratification and TAVI is reimbursed only for patients at a high operative risk in the Netherlands, 18% of Dutch patients undergoing TAVI are younger than 75 years [4].

**Fig. 1:**
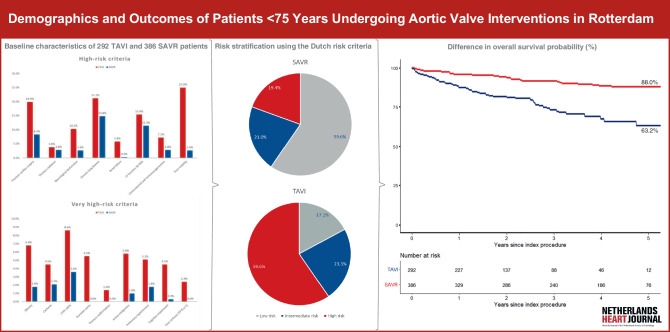
Infographic: Demographics and outcomes of patients < 75 years of age undergoing aortic valve interventions in Rotterdam. *TAVI* transcatheter aortic valve implantation, *SAVR* surgical aortic valve replacement, *LV* left ventricular, *LVEF* left ventricular ejection fraction, *CP* Child-Pugh class

In 2020, the Dutch Society of Cardiology and the Dutch Cardiothoracic Surgeons Society jointly formulated risk stratification criteria to harmonise and facilitate the multidisciplinary heart team (MHT) decision-making process for TAVI or surgical aortic valve replacement (SAVR) [5, 6]. We aimed to (1) apply these risk criteria to define risk phenotypes that may clarify why patients aged < 75 years would undergo TAVI instead of SAVR, and (2) report on the clinical outcome of patients < 75 years old who undergo SAVR and TAVI (Infographic: Fig. 1).

## Methods

### Design

This study included all consecutive patients aged 50–75 years who underwent SAVR or TAVI at the Erasmus University Medical Centre between 2015 and 2020 for severe AS, mixed aortic valve disease or failed bioprosthesis, excluding active endocarditis. Both isolated and combined procedures were included, and for SAVR patients both mechanical prostheses and bioprostheses were included. All study procedures were in accordance with the Declaration of Helsinki and the study did not fall under the scope of the Medical Research Involving Human Subjects Act per institutional review boards review (MEC-2021-0600).

### Data collection

Data were captured in a dedicated database as part of a national collaboration programme among Dutch heart centres aiming to improve quality of care in patients undergoing transcatheter heart valve interventions and cardiac surgery. Mortality data were collected by consulting municipality registration data.

### Risk criteria and cluster variable definitions

**Fig. 2:**
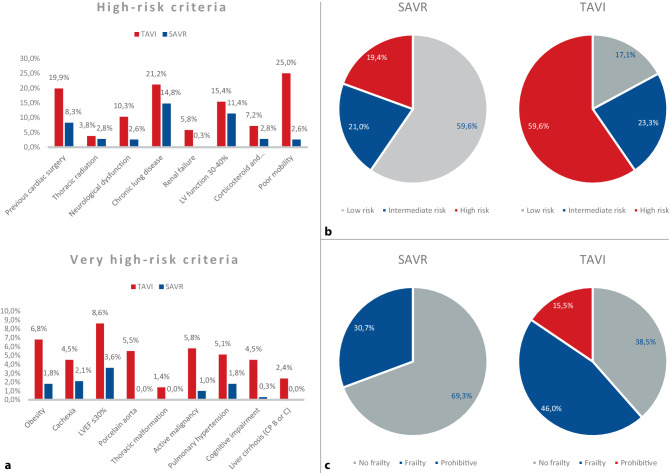
**a** Distribution of risk criteria. **b** Distribution of risk categories. *Low risk* no risk criteria, *Intermediate risk* 1 high-risk criterion, *High risk* ≥ 2 high-risk criteria or ≥ 1 very high-risk criteria. **c** Distribution of frailty. *TAVI* transcatheter aortic valve implantation, *SAVR* surgical aortic valve replacement, *LV* left ventricular, *LVEF* left ventricular ejection fraction, *CP* Child-Pugh class

The high-risk and very high-risk criteria are shown in Fig. 2a. Patients were classified as low risk if they met none of the risk criteria; intermediate risk if they met one high-risk criterion, or high risk if they met two or more high-risk criteria or at least one very high-risk criterion. TAVI patients were discussed by the MHT and may have been deemed at higher operative risk because of variables not captured by the Dutch risk criteria. A proxy variable was used for frailty, consisting of cognitive impairment, poor mobility, previous stroke, neurological dysfunction and body mass index < 20.

To analyse the impact of baseline characteristics and risk criteria on outcome, variables were clustered based on clinical considerations. Variables within a cluster were considered equally important and patients with ≥ 1 of the characteristics that formed a cluster of variables were deemed positive for that cluster. The following clusters were defined: (1) demographics, (2) cardiovascular comorbidities, (3) non-cardiovascular comorbidities, (4) procedural characteristics, (5) frailty, (6) impediment to surgery, and (7) a cluster of miscellaneous comorbidities (Table S1, Electronic Supplementary Material). The primary endpoint was all-cause mortality.

### Statistical analysis

Continuous data were tested for normality by means of Shapiro-Wilk test, presented as mean and standard deviation or median and 25th–75th percentiles, depending on distribution, and compared between groups using independent sample *t*-tests or Mann-Whitney U tests, as appropriate. Categorical variables are presented as counts and percentages and were compared between groups using a chi-square test or Fisher’s exact test. Mortality is presented as Kaplan-Meier estimated percentages. Associations between treatment group and the primary outcome were investigated by Cox proportional hazard regression analyses. Interactions between risk-category subgroups and treatment group, and interactions between baseline characteristics and treatment group were tested by adding product terms of risk category and treatment group and product terms of the clustered variables and treatment group to the respective Cox regression models. Relations between cluster variables and the primary outcome were tested in simple (including only the variable of interest and the grouping variable) and complex Cox models, stratified for treatment group. Variables with a *p*-value < 0.10 or a *p*_interaction_ < 0.10 in the simple model were included in the complex model. The proportional hazards assumption was tested for all variables by using Schoenfeld residuals tests.

All analyses were performed in Statistics Pack for Social Sciences version 28.0.0.0 (IBM Corp., Armonk, NY, USA) and R version 4.2.1. A two-tailed *p*-value of < 0.05 was considered statistically significant.

## Results

### Demographics

A total of 678 patients were included. Baseline characteristics are shown in Tab. 1. Median age was 69 years (64–72), and 439 patients (64.7%) were male. Median EuroSCORE II was 1.8% (1.1–3.2%).

**Table 1:** Patient demographics and procedural characteristics

Demographics	Overall (*n* = 678)	TAVI (*n* = 292)	SAVR (*n* = 386)	*p*-Value
Age (years), median (IQR)	69 (64–72)	70 (67–72)	68 (63–71)	< 0.001
Gender, male (%)	439 (64.7)	184 (63.0%)	255 (66.1%)	0.411
BMI (kg/m^2)^, median (IQR)	28.0 (24.8–31.9)	28.1 (24.5–33.0)	28.0 (25.0–31.3)	0.321
LVEF, median (IQR)	55% (50–60)	55% (45–60)	55% (55–60)	0.001
LVEF < 50%, counts (%)	165 (24.3%)	93 (31.8%)	72 (18.7%)	< 0.001
eGFR (Cockcroft-Gault) (ml/min), median (IQR)	80.2 (63.8–96.8)	76.8 (55.7–95.2)	82.2 (67.9–99.1)	< 0.001
eGFR < 60 ml/min (%)	134 (19.8%)	85 (29.1%)	49 (12.7%)	< 0.001
eGFR < 30 ml/min (%)	20 (2.9%)	17 (5.8%)	3 (0.8%)	< 0.001
Diabetes,* n* (%)	221 (32.6%)	119 (40.8%)	102 (26.4%)	< 0.001
Atrial fibrillation, *n* (%)	132 (19.5%)	77 (26.4%)	55 (14.2%)	< 0.001
Previous stroke, *n* (%)	50 (7.4%)	26 (8.9%)	24 (6.2%)	0.185
Previous PCI, *n* (%)	110 (16.2%)	71 (24.3%)	39 (10.1%)	< 0.001
Previous CABG, *n* (%)	57 (8.4%)	49 (16.8%)	8 (2.1%)	< 0.001
Previous aortic valve intervention including BAV, *n* (%)	36 (5.3%)	20 (6.8%)	16 (4.1%)	0.12
*Indication AVR, n (%)*				0.52
– Severe AS	644 (95.0%)	280 (95.9%)	364 (94.3%)	
– Mixed AV disease	11 (1.6%)	3 (1%)	8 (2.1%)	
– Prosthesis failure	23 (3.4%)	9 (3.1%)	14 (3.6%)	
*Procedural characteristics*				
*Surgical procedure, n* (%)				
– Isolated AVR	–	–	231 (59.8%)	
– AVR and CAB	–	–	130 (33.7%)	
– AVR and other valve surgery (**±** CAB)	–	–	25 (6.5%)	
*TAVI procedure, n (%)*				
– Isolated TAVI	–	238 (81.5%)	–	
– Staged TAVI and PCI	–	21 (7.2%)	–	
– Concomitant PCI	–	33 (11.3%)	–	
*AVR prosthesis type, n (%)*				< 0.001
– Bioprosthesis	–	292 (100%)	295 (76.4%)	
– Mechanoprosthesis	–		91 (23.6%)	
Euroscore II (IQR)	1.8% (1.1–3.2)	2.2% (1.3–4.9)	1.6% (1.0–2.7)	< 0.001
*Risk profile, counts* (%)				< 0.001
– Low risk	280 (41.3%)	50 (17.1%)	230 (59.6%)	
– Intermediate risk	149 (22.0%)	68 (23.3%)	81 (21.0%)	
– High risk	249 (36.7%)	174 (59.6%)	75 (19.4%)	

*TAVI* transcatheter aortic valve implantation, *SAVR* surgical aortic valve replacement, *IQR* interquartile range, *BMI* body mass index, *LVEF* left ventricular ejection fraction, *eGFR* estimated glomerular filtration rate, *PCI* percutaneous coronary intervention, *CABG* coronary artery bypass graft, *BAV* balloon aortic valvuloplasty, *AS* aortic stenosis

Of the patients included, 292 underwent TAVI and 386 underwent SAVR. There was no significant sex difference between groups (63.0% vs 66.1% male in TAVI vs SAVR; *p* = 0.14). TAVI patients were older (70 vs 68 years, *p* < 0.001). More TAVI patients had a left ventricular ejection fraction (LVEF) < 50% (31.8% vs 18.7%; *p* < 0.001), diabetes (40.8% vs 26.4%; *p* < 0.001), atrial fibrillation (26.4% vs 14.2%; *p* < 0.001) or chronic kidney disease (estimated glomerular filtration rate < 60 ml/min in 29.1% vs 12.7%; *p* < 0.001). EuroSCORE II was higher in the TAVI population (2.2 vs 1.6%, *p* < 0.001).

The indication for valve intervention was AS in 95.0% of patients, with no significant difference between TAVI and SAVR (*p* = 0.52). Median length of hospital stay for TAVI patients was 4 days (2–7) vs 5 days (3–6, *p* < 0.001) for SAVR patients. SAVR was combined with coronary artery bypass surgery or other valve surgery in 40.2% of patients; TAVI was combined with percutaneous coronary intervention in 11.3% of patients.

### Risk profile

Risk criteria distribution over the two cohorts is illustrated in Fig. 2a. Notably, six of the eight high-risk criteria and eight of the nine very high-risk criteria were significantly more prevalent in the TAVI group.

The risk profile distribution differed significantly between TAVI and SAVR patients (*p* < 0.001) (Fig. 2b). Of note is that 174 TAVI patients (59.6%) were at high risk based on the Dutch risk criteria as opposed to 75 SAVR patients (19.4%). Conversely, 230 SAVR patients (59.6%) versus 50 TAVI patients (17.1%) were deemed low risk.

TAVI and SAVR patients differed significantly within risk cohorts (Table S2, Electronic Supplementary Material). Across risk-category subgroups, TAVI patients were older than SAVR patients. Low-risk patients undergoing TAVI had more non-cardiovascular comorbidities (46.0% vs 30.0%, *p* = 0.029) and peripheral artery disease (20.0% vs 7.8%, *p* = 0.009) and were more often female (48% vs 29.6%, *p* *=* 0.012) compared to low-risk SAVR patients. High-risk TAVI patients were more often frail (50.6% vs 30.7%, *p* = 0.004) or had peripheral artery disease (38.5% vs 12.0%, *p* < 0.001). Of the high-risk TAVI patients, 15.5% were judged to be at prohibitive risk, defined by the presence of a porcelain aorta, a thoracic malformation or Child-Pugh class B or C liver cirrhosis (Fig. 2c).

### All-cause mortality

**Fig. 3:**
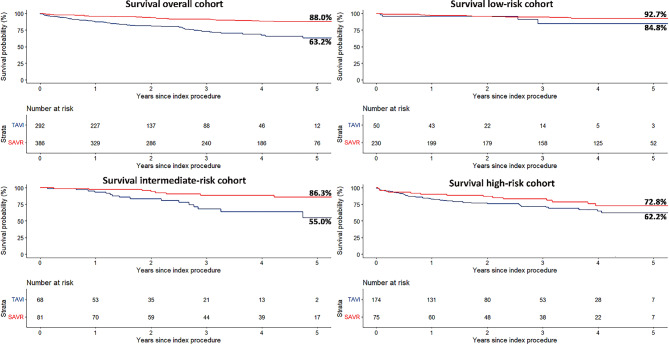
Kaplan-Meier curves of all-cause survival. *TAVI* transcatheter aortic valve implantation, *SAVR* surgical aortic valve replacement

The Kaplan-Meier survival percentages and the results of the Cox proportional hazards regression model are summarised in Table S3, Electronic Supplementary Material. There was no statistically significant difference in 30-day all-cause mortality between TAVI and SAVR patients (2.4% vs 0.8%, *p* = 0.083). Mortality at 1 year was higher for TAVI than for SAVR patients (12.5% vs 4.3%, *p* < 0.001), as was mortality at 5 years (36.8% vs 12.0%, *p* < 0.001) (Fig. 3).

There were numerical differences between low-, intermediate- and high-risk patients, but no evidence for interaction between risk category and treatment group (*p*_interaction_ = 0.30 for low risk vs intermediate risk; *p*_interaction_ = 0.93 for low risk vs high risk). Hence, any statistical differences found may be the consequence of small treatment groups. The overall hazard ratio (HR) for TAVI vs SAVR was 3.3 [95% confidence interval (95% CI): 2.2–4.9; *p* < 0.001].

### Cluster variable analysis

Prevalence of all cluster variables except demographics differed significantly between treatment groups (Table S4, Electronic Supplementary Material). Tab. 2 shows the simple and complex models of the 5‑year Cox regression analysis for all-cause mortality. The simple model suggests an increase in hazard of mortality from the cardiovascular comorbidities cluster [HR 1.7 (95% CI: 1.2–2.6)], the non-cardiovascular comorbidities cluster [HR 1.7 (95% CI: 1.07–2.6)] and the cluster of miscellaneous conditions [HR 4.2 (95% CI: 2.6–6.6)]. An isolated procedure was associated with a reduced hazard [HR 0.60 (95% CI: 0.38–0.95)]. No significant differences in HR were noted between TAVI and SAVR patients (*p*_interaction_ > 0.05 for all cluster variables). In the complex model, the cardiovascular comorbidities cluster [HR 1.6 (95% CI: 1.1–2.4)], isolated procedure [HR 0.55 (95% CI: 0.34–0.91)] and the cluster of miscellaneous conditions [HR 4.5 (95% CI: 2.8–7.4)] were independently associated with mortality at 5 years. For SAVR patients, the cluster of impediments to surgery also conveyed an increase in hazard of mortality.

**Table 2:** Simple and complex Cox regression analysis for 5‑year all-cause mortality

					Model for interaction		
	TAVI		SAVR			Overall (stratified by treatment)	
	HR (95% CI)	*p*-value	HR (95% CI)	*p*-value	*p*-value of interaction between TAVR and SAVR	HR (95% CI)	*p*-value
**Simple model**							
*Demographics*							
– Male gender	1.1 (0.70–2.0)	0.538	1.2 (0.61–2.3)	0.595	0.979	1.2 (0.78–1.8)	0.424
– Age < 65 years	1.7 (0.97–2.9)	0.066	0.69 (0.34–1.4)	0.319	0.056	1.2 (0.75–1.8)	0.485
*Cluster cardiovascular*	1.5 (0.91–2.4)	0.109	2.3 (1.2–4.5)	0.014	0.285	1.7 (1.2–2.6)	0.006
– LVEF < 30%							
– Atrial fibrillation							
– Systolic pulmonary pressure > 55 mm Hg							
*Cluster non-cardiovascular comorbidities*	1.3 (0.72–2.2)	0.419	2.3 (1.2–4.4)	0.012	0.172	1.7 (1.07–2.6)	0.024
– Obesity							
– Diabetes							
– Renal function < 60 ml/min							
– Chronic lung disease							
*Cluster procedural*	0.67 (0.33–1.4)	0.262	0.55 (0.29–1.02)	0.061	0.664	0.60 (0.38–0.95)	0.030
– Isolated procedure							
*Cluster frailty*	0.84 (0.50–1.4)	0.530	1.2 (0.44–3.5)	0.675	0.505	0.90 (0.56–1.5)	0.675
– Cognitive impairment							
– Neurological dysfunction							
– Poor mobility							
– BMI < 20 kg/m^2^							
– Previous stroke							
*Cluster surgical impediment*	1.1 (0.68–1.8)	0.697	2.2 (1.1–4.1)	0.021	0.102	1.4 (0.93–2.1)	0.111
– Previous sternotomy							
– Porcelain aorta							
– Peripheral vascular disease							
– Thoracic radiation							
– Thoracic malformation							
*Cluster miscellaneous*	4.5 (2.7–7.8)	< 0.001	2.7 (0.94–7.5)	0.064	0.353	4.2 (2.6–6.6)	< 0.001
– Active malignancy							
– Liver cirrhosis							
– Immunocompromised status							
**Complex model**							
*Cluster demographics*							
– Male gender						–	–
– Age < 65 years						TAVR: 1.5 (0.87–2.7) SAVR: 0.68 (0.33–1.4)	0.144 0.305
*Cluster cardiovascular*						1.6 (1.1–2.4)	0.021
*Cluster non-cardiovascular*						1.4 (0.91–2.2)	0.127
*Cluster procedural*						0.55 (0.34–0.91)	0.018
*Cluster surgical impediment*						TAVR: 1.4 (0.85–2.3) SAVR: 1.9 (1.0–3.7)	0.178 0.048
*Cluster miscellaneous*						4.5 (2.8–7.4)	< 0.001

*TAVI* transcatheter aortic valve implantation, *SAVR* surgical aortic valve replacement, *HR* hazard ratio, *95% CI* 95% confidence interval, *LVEF* left ventricular ejection fraction, *BMI* body mass index

## Discussion

This study investigated the differences in risk phenotype and outcomes of TAVI and SAVR patients aged below 75 years. The main findings of this study are:

1. In patients aged < 75 years, there is a distinct risk profile difference between TAVI and SAVR patients.
2. Overall, TAVI patients showed higher 1‑year and 5‑year mortality than SAVR patients, which may be explained by the overall higher prevalence of high-risk characteristics in the TAVI group.
3. Five-year mortality was impacted most by active malignancy, liver cirrhosis and immunocompromised status.
4. Application of the Dutch risk criteria was only partially effective in determining a patient’s actual risk, which validates the guideline-recommended complementary contribution of clinical judgement by the local MHT.

Our study specifically focused on patients < 75 years of age who required aortic valve intervention. The Euroscore II score was 2.2% for the TAVI cohort and 1.6% for the SAVR cohort, which compares with a 1.5% EuroScore II in the PARTNER 3 trial [2]. TAVI patients were notably different from SAVR patients in terms of risk profile. Most TAVI patients had high-risk criteria, whereas 59.6% of SAVR patients had no such criteria. This may explain the higher 5‑year mortality with TAVI (36.8%) than with SAVR (12.0%). TAVI 1‑year mortality and 5‑year mortality is in keeping with the 12.3% and 39.2% reported in the PARTNER 2 trial [7, 8], which included an intermediate- to higher-risk population. SAVR 1‑year mortality follows the rates in the lower-risk SURTAVI (6.8%), PARTNER 3 (2.5%) and Evolut Low Risk (3.0%) trials [2, 3, 9]. SAVR 5‑year mortality, however, is much lower than reported in previous intermediate-risk trials, but slightly higher than reported in the PARTNER 3 5‑year results [10]. Of note is that patients in our study were different and much younger than the patients in the randomised trials.

Our mortality findings reinforce the higher risk profile of TAVI patients than of SAVR patients that seems difficult to unveil with contemporary risk algorithms, including the Dutch risk criteria. The absence of a statistically significant difference in overall outcome within the respective risk strata for TAVI and SAVR may suggest appropriate risk assessment and consequent treatment allocation by the MHT, but the small sample sizes of the separate risk groups limit definite conclusions. Moreover, numerical outcome differences between TAVI and SAVR should be interpreted with the recognition of remaining unaccountable confounders that may drive a MHT to select one therapy over the other.

We performed an analysis of clustered variables to understand the clinical impact of a set of variables. The presence of an active malignancy, liver cirrhosis or the use of immunomodulatory drugs was associated with a 4.5 times higher risk for 5‑year mortality, whereas cardiovascular comorbidities resulted in a 1.6 times higher risk. Of note is that isolated procedures were much more frequent in the TAVI cohort than in the SAVR cohort and were found to be associated with decreased mortality at 5 years. As expected, the cluster of impediments to surgery that included presence of a hostile chest and previous cardiac surgery affected the outcome after SAVR but not after TAVI, clearly confirming that such patients fare better with TAVI than with SAVR.

Our findings contrast with registry data that included older patients, primarily focused on in-hospital and 30-day mortality and identified symptoms/urgency, pulmonary hypertension and impaired LVEF as the main predictors for short-term mortality [11–14]. Of these, the German registry investigating the novel Aortic Valve Score included 11,794 patients that underwent an aortic valve intervention in 2008, of whom 634 were TAVI patients [14]. Sixty-one percent of patients were < 75 years old. Mortality was 3.4% for SAVR patients and 10.6% for TAVI patients, similar to our findings. In addition to cardiovascular disease, non-cardiovascular comorbidities, peripheral arterial disease and prior sternotomy also correlated with all-cause mortality.

Our study highlights the fact that dynamic and individualised risk assessment by the MHT is difficult, if not impossible, to capture with a static risk score algorithm. In the context of the Dutch reimbursement system, younger patients (< 75 years old) who are referred for TAVI remain at higher (operative) risk for mortality than those who undergo SAVR.

### Limitations

Our study is inherently limited by its non-randomised and retrospective design and relatively small sample size, especially in the subgroup cohorts stratified by risk-category. Variables that are collected in the Dutch heart centre collaboration are elaborate but not equal for TAVI and SAVR. Extensive efforts were performed to collect missing data through patient chart review. Frailty was not systematically reported and therefore a proxy variable was applied. Treatment allocation was at the discretion of the heart team and, although we aimed to collect all relevant variables required for this decision, some uncollected subjective measures or unmeasured characteristics may have been vital in the final decision-making process.

We included patients who required SAVR or TAVI for severe AS, mixed aortic valve disease or bioprosthetic valve failure. In the SAVR cohort, the relative proportion of mixed aortic valve disease and bioprosthetic valve failure was higher and both biological and mechanical prostheses were allowed. This should be acknowledged when comparing TAVI and SAVR patients in our study and putting our data in perspective compared with other reports that exclusively included AS patients.

## Conclusion

At the Erasmus University Medical Centre, in patients aged < 75 years, TAVI is selected for higher-risk phenotypes and overall has higher long-term mortality than SAVR. There was no evidence for a difference in survival after SAVR and TAVI for patients in similar risk strata. Individualised patient risk assessment requires MHT involvement.

## Supplementary Information

Supplementary table S1

Supplementary table 2 Patient demographics of low-, intermediate- and high-risk patient subgroups

Supplementary table 3 Kaplan-Meier survival estimates and Cox regression

Supplementary table 4 Frequencies of individual values of clustered variables
