# Supplementary material for: Demographics and outcomes of patients younger than 75 years undergoing aortic valve interventions in Rotterdam
Source: Neth Heart J. 2024 Aug 20;32(10):348–55. doi: 10.1007/s12471-024-01888-2 (PMC11413251; doi:10.1007/s12471-024-01888-2)
Supplement: Supplementary file 1 — Supplementary table S1 [file 12471_2024_1888_MOESM1_ESM.docx]

**Supplementary table S1**

| Cluster name | Included characteristics |
| --- | --- |
| 1. Demographics | Age < 65 years, male gender |
| 1. Cardiovascular comorbidity | Poor LVEF (<30%), pulmonary hypertension (systolic pulmonary artery pressure ≥55mmHg), and history of atrial fibrillation |
| 1. Non-cardiovascular comorbidity | Obesity, diabetes, renal function <60mL/min and chronic lung disease |
| 1. Procedural | Isolated procedure |
| 1. Frailty | Cognitive impairment, poor mobility, previous stroke, neurological dysfunction and BMI<20 |
| 1. Surgical impediment | Thoracic deformation, arterial vascular pathology (including aortic aneurysms, previous carotid surgery or severe femoral artery disease), porcelain aorta, history of thoracic radiation and previous cardiac surgery |
| 1. Miscellaneous comorbidities | Active malignancy, liver cirrhosis (Child-Pugh B or C) and immunocompromised status (defined as chronic use of immunomodulatory drugs) |
