# Supplementary material for: Demographics and outcomes of patients younger than 75 years undergoing aortic valve interventions in Rotterdam
Source: Neth Heart J. 2024 Aug 20;32(10):348–55. doi: 10.1007/s12471-024-01888-2 (PMC11413251; doi:10.1007/s12471-024-01888-2)
Supplement: Supplementary file 2 — Supplementary table 2 Patient demographics of low-, intermediate- and high-risk patient subgroups [file 12471_2024_1888_MOESM2_ESM.docx]

**Supplementary table 2. Patient demographics of low-, intermediate- and high-risk patient subgroups.**

|  | Low risk | | | | Intermediate risk | | | | High risk | | | |
| --- | --- | --- | --- | --- | --- | --- | --- | --- | --- | --- | --- | --- |
|  | **Overall (n=280)** | **TAVI**  **(n = 50)** | **SAVR (n=230)** | **p-value** | **Overall (n=149)** | **TAVI**  **(n = 68)** | **SAVR (n=81)** | **p-value** | **Overall (n=249)** | **TAVI**  **(n = 174)** | **SAVR (n=75)** | **p-value** |
| Demographics |  |  |  |  |  |  |  |  |  |  |  |  |
| Age, median (IQR) | 69 (64-72) | 70.5 years (68.8-73) | 68 years (64-71.3) | 0.002 | 69 (65-72) | 70.0 years (67.0-72) | 68 years (64.0-71.0) | 0.033 | 69 (64-72) | 70. years (65-72) | 66 years (60-71.0) | <0.001 |
| Gender, male (%) | 198 (67.1%) | 26 (52.0%) | 162 (70.4%) | 0.012 | 92 (61.7%) | 41 (60.3%) | 51 (63.0%) | 0.738 | 159 (63.9%) | 17 (67.2%) | 42 (56.0%) | 0.09 |
| BMI, median (IQR) | 28.2 (25.3-31.5) | 28.6 kg/m^2^ (24.7-31.5) | 28.1 kg/m^2^ (25.4-31.5) | 0.922 | 27.9 (25.1-31.1) | 27.64kg/m^2^ (24.4-31.4) | 28.4 kg/m^2^ (26.1-31.1) | 0.356 | 27.9 (23.4-32.3) | 29.0 kg/m^2^ (24.0-33.9) | 26.3 kg/m^2^ (22.6-30.8) | 0.018 |
| LVEF, median (IQR) | 60% (55-60) | 55% (53.8-60) | 60% (55-60) | 0.073 | 55% (50-60) | 55% (50.3-60.0) | 55% (50.5-60.0) | 0.403 | 50% (38.5-55) | 52% (38-58.0) | 40% (35-55) | 0.023 |
| LVEF <50%, counts (%) | 17 (6.1%) | 6 (12.0%) | 11 (4.8%) | 0.053 | 31 (20.8%) | 13 (19.1%) | 18 (22.3%) | 0.642 | 117 (47.0%) | 74 (42.5%) | 43 (57.3%) | 0.032 |
| eGFR (cockcroft-Gault), median (IQR) | 84.3 ml/min (68.6-99.4) | 85.4 ml/min (69.2-97.2) | 84.0 ml/min (68.8-100.2) | 0.612 | 80.7 ml/min (63.1-96.2) | 75.6 ml/min (55.4-91.7) | 84.1 ml/min (67.4-100.1) | 0.018 | 76.0 ml/min (54.0-94.1) | 74.0 ml/min (53.1-95.4) | 77.7 ml/min (63.4-93.1) | 0.534 |
| eGFR <60ml/min (%) | 32 (11.4%) | 7 (14.0%) | 25 (10.9%) | 0.528 | 28 (18.8%) | 21 (30.9%) | 7 (8.6%) | <0.001 | 74 (29.7%) | 57 (32.8%) | 17 (22.7%) | 0.11 |
| eGFR <30ml/min (%) | 0 (0.0%) | 0 (0.0%) | 0 (0.0%) | - | 4 (2.7%) | 4 (5.9%) | 0 (0.0%) | 0.041 | 16 (6.4%) | 13 (7.5%) | 3 (4.0%) | 0.405 |
| Diabetes, counts (%) | 75 (24.6) | 18 (36.0%) | 51 (22.2%) | 0.04 | 45 (30.2%) | 19 (27.9%) | 26 (32.1%) | 0.582 | 107 (43.0%) | 82 (47.1%) | 25 (33.3%) | 0.044 |
| Atrial fibrillation (%) | 34 (12.1%) | 9 (18.0%) | 25 (10.9%) | 0.162 | 30 (20.1%) | 18 (26.5%) | 12 (14.8%) | 0.077 | 68 (27.3%) | 50 (28.7%) | 18 (24.0%) | 0.442 |
| Previous stroke (%) | 0 (0%) | 0 (0.0%) | 0 (0.0%) | - | 15 (10.1%) | 3 (4.4%) | 12 (14.8%) | 0.053 | 35 (14.1%) | 23 (13.2%) | 12 (16.0%) | 0.562 |
| Previous PCI (%) | 25 (8.9%) | 7 (14.0%) | 18 (7.8%) | 0.165 | 33 (22.1%) | 23 (33.8%) | 10 (12.3%) | 0.002 | 52 (20.9%) | 41 (23.6%) | 11 (14.7%) | 0.113 |
| Previous CABG (%) | 0 (0%) | 0 (0.0%) | 0 (0.0%) | - | 22 (14.8%) | 16 (23.5%) | 6 (7.4%) | 0.006 | 35 (14.1%) | 33 (19.0%) | 2 (2.7%) | <0.001 |
| Previous aortic valve intervention (%) | 0 (0%) | 0 (0.0%) | 0 (0.0%) | - | 12 (8.1%) | 4 (5.9%) | 8 (9.9%) | 0.372 | 24 (9.6%) | 16 (9.2%) | 8 (10.7%) | 0.718 |
| Indication AVR |  |  |  | 0.939 |  |  |  | 0.433 |  |  |  | 0.104 |
| Severe AS | 274 (97.9%) | 49 (98.0%) | 225 (97.8%) |  | 138 (92.6%) | 65 (95.6%) | 73 (90.1%) |  | 232 (93.2%) | 166 (95.4%) | 66 (88.0%) |  |
| Mixed AV disease | 6 (2.1% | 1 (2.0%) | 5 (2.2%) |  | 3 (2.0%) | 1 (1.5%) | 2 (2.5%) |  | 2 (0.8%) | 1 (0.6%) | 1 (1.3%) |  |
| Prosthesis failure | 0 (0.0%) | 0 (0.0%) | 0 (0.0%) |  | 8 (5.4%) | 2 (2.9%) | 6 (7.4%) |  | 15 (6.0%) | 7 (4.0% | 8 (10.7%) |  |
|  |  |  |  |  |  |  |  |  |  |  |  |  |
| Procedural characteristics |  |  |  |  |  |  |  |  |  |  |  |  |
| Isolated AVR | 192 (68.6%) | 43 (86.0%) | 149 (64.8%) | 0.003 | 102 (68.5%) | 61 (89.7%) | 41 (50.6%) | <0.001 | 196 (78.7%) | 174 (100%) | 41 (54.7%) | <0.001 |
| AVR prosthesis type |  |  |  | <0.001 |  |  |  | <0.001 |  |  |  | <0.001 |
| Bioprosthesis | 236 (84.3%) | 50 (100%) | 186 (80.9%) |  | 130 (87.2%) | 68 (100%) | 62 (76.5%) |  | 221 (88.8%) | 174 (100%) | 47 (62.7%) |  |
| Mechanoprosthesis | 44 (15.7%) |  | 44 (19.1%) |  | 17 (11.6) |  | 17 (23.5%) |  | 28 (11.9%) |  | 28 (37.3%) |  |
| Euroscore II (IQR) | 1.3% (0.96-1.8) | 1.3% (1.1-1.6) | 1.3% (0.94-1.8) | 0.471 | 2.3% (1.3-4.0) | 2.2% (1.3-4.3) | 2.5% (1.3-3.9) | 0.894 | 2.7% (1.6-5.8) | 2.6% (1.6-6.0) | 2.9% (1.4-5.6) | 0.901 |
