# Supplementary material for: Demographics and outcomes of patients younger than 75 years undergoing aortic valve interventions in Rotterdam
Source: Neth Heart J. 2024 Aug 20;32(10):348–55. doi: 10.1007/s12471-024-01888-2 (PMC11413251; doi:10.1007/s12471-024-01888-2)
Supplement: Supplementary file 3 — Supplementary table 3 Kaplan-Meier survival estimates and Cox regression [file 12471_2024_1888_MOESM3_ESM.docx]

**Table S3** Kaplan-Meier survival estimates and Cox regression

|  | TAVI | SAVR | Cox regression  (for TAVR vs SAVR) | | *p*_interaction_ for risk group vs treatment group |
| --- | --- | --- | --- | --- | --- |
|  | Kaplan-Meier survival | Kaplan-Meier survival | HR (95% CI) | *p*-value |  |
| *Overall* |  |  |  |  |  |
| 30 days | 2.4% (0.6-4.2%) | 0.8% (0-1.7%) |  |  |  |
| 1 year | 12.5% (8.5-16.3%) | 4.3% (2.2-6.4%) |  |  |  |
| 5 years | 36.8% (26.7-45.4%) | 12.0% (8.2-15.7%) | 3.3 (2.2-4.9) | <0.001 |  |
| *Low risk* |  |  |  |  | Reference |
| 30 days | 2.0% (0-5.9%) | 0% |  |  |  |
| 1 year | 4.1% (0-9.5%) | 3.2% (0.8-5.6%) |  |  |  |
| 5 years | 15.2% (0-29.2%) | 7.3% (3.5-10.9%) | 1.8 (0.59-5.5) | 0.300 |  |
| *Intermediate risk* |  |  |  |  | 0.30 |
| 30 days | 0% | 0% |  |  |  |
| 1 year | 6.6% (0-12.6%) | 2.6% (0-6.1%) |  |  |  |
| 5 years | 45.0% (18.8-62.8%) | 13.7% (4.1-22.4%) | 3.4 (1.4-8.1) | 0.005 |  |
| *High risk* |  |  |  |  | 0.93 |
| 30 days | 3.5% (0.7-6.1%) | 4.0 (0-8.4%) |  |  |  |
| 1 year | 17.2% (11.3-22.7%) | 9.6% (2.6-16.1%) |  |  |  |
| 5 years | 37.8% (26.2-47.5%) | 27.2% (13.4-38.8%) | 1.6 (0.89-2.9) | 0.118 |  |
|  |  |  |  |  |  |

*TAVI* transcatheter aortic valve implantation, *SAVR* surgical aortic valve replacement, *HR* hazard ratio, *95%* *CI* 95% confidence interval
