# Supplementary material for: Demographics and outcomes of patients younger than 75 years undergoing aortic valve interventions in Rotterdam
Source: Neth Heart J. 2024 Aug 20;32(10):348–55. doi: 10.1007/s12471-024-01888-2 (PMC11413251; doi:10.1007/s12471-024-01888-2)
Supplement: Supplementary file 4 — Supplementary table 4 Frequencies of individual values of clustered variables [file 12471_2024_1888_MOESM4_ESM.docx]

**Supplementary table 4. Frequencies of individual values of clustered variables.**

|  | Counts TAVI | Counts SAVR | p-value |
| --- | --- | --- | --- |
| Demographics | **210 (71.9%)** | **300 (77.7%)** | **0.083** |
| Male gender | 184 (63.0%) | 255 (66.1%) | 0.411 |
| Age < 65 years | 56 (19.2%) | 125 (32.4%) | <0.001 |
| Cluster Cardiovascular | **97 (33.2%)** | **68 (17.6%)** | **<0.001** |
| LVEF < 30% | 25 (8.6%) | 14 (3.6%) | 0.006 |
| Atrial fibrillation | 77 (26.4%) | 55 (14.2%) | <0.001 |
| Systolic pulmonary pressure > 55mmHg | 15 (5.1%) | 7 (1.8%) | 0.016 |
| Cluster non-cardiovascular comorbidities | **205 (70.2%)** | **168 (43.5%)** | **<0.001** |
| Obesity | 20 (6.8%) | 7 (1.8%) | <0.001 |
| Diabetes | 119 (40.8%) | 102 (26.4%) | <0.001 |
| Renal function < 60ml/min | 85 (29.1%) | 49 (12.7%) | <0.001 |
| Chronic lung disease | 62 (21.2%) | 57 (14.8%) | 0.028 |
| Cluster procedural |  |  |  |
| Isolated procedure | 259 (88.7%) | 231 (59.8%) | <0.001 |
| Cluster frailty | **106 (36.4%)** | **36 (9.3%)** | **<0.001** |
| Cognitive impairment | 13 (4.5%) | 1 (0.3%) | <0.001 |
| Neurologic dysfunction | 30 (10.3%) | 10 (2.6%) | <0.001 |
| Poor mobility | 73 (25.0%) | 10 (2.6%) | <0.001 |
| BMI < 20kg/m^2^ | 13 (4.5%) | 8 (2.1%) | 0.077 |
| Previous stroke | 26 (8.9%) | 24 (6.2%) | 0.185 |
| Cluster surgical impediment | **143 (49.0%)** | **84 (21.8%)** | **<0.001** |
| Previous sternotomy | 58 (19.9%) | 32 (8.3%) | <0.001 |
| Porcelain aorta | 16 (5.5%) | 0 | <0.001 |
| Peripheral vascular disease | 95 (32.5%) | 41 (10.6%) | <0.001 |
| Thoracic radiation | 11 (3.8%) | 11 (2.8%) | 0.36 |
| Thoracic malformation | 4 (1.4%) | 0 | 0.006 |
| Cluster miscellaneous | **43 (14.7%)** | **17 (4.4%)** | **<0.001** |
| Active malignancy | 17 (5.8%) | 4 (1.0%) | <0.001 |
| Liver cirrhosis | 7 (2.4%) | 0 | 0.02 |
| Immunocompromised status | 21 (7.2%) | 11 (2.8%) | 0.01 |
